# In Vivo Myelin Water Quantification Using Diffusion–Relaxation Correlation MRI: A Comparison of 1D and 2D Methods

**DOI:** 10.1007/s00723-023-01584-1

**Published:** 2023-08-04

**Authors:** Sebastian Endt, Maria Engel, Emanuele Naldi, Rodolfo Assereto, Malwina Molendowska, Lars Mueller, Claudio Mayrink Verdun, Carolin M. Pirkl, Marco Palombo, Derek K. Jones, Marion I. Menzel

**Affiliations:** 1 Technical University of Munich, Munich, Germany; 2 AImotion Bavaria, Technische Hochschule Ingolstadt, Ingolstadt, Germany; 3 Cardiff University Brain Research Imaging Centre (CUBRIC), Cardiff University, Cardiff, United Kingdom; 4 Technische Universität Braunschweig, Braunschweig, Germany; 5 Universität Graz, Graz, Austria; 6 Medical Radiation Physics, Lund University, Lund, Sweden; 7 Biomedical Imaging Science Department, Leeds Institute of Cardiovascular and Metabolic Medicine (LICAMM), University of Leeds, Leeds, United Kingdom; 8 Munich Center for Machine Learning, Munich, Germany; 9 GE HealthCare, Munich, Germany

**Keywords:** MWF mapping, Microstructure, Relaxometry, Diffusometry, Multi-component, Multidimensional, Multi-exponential, Multiparametric, Correlation imaging

## Abstract

Multidimensional Magnetic Resonance Imaging (MRI) is a versatile tool for microstructure mapping. We use a diffusion weighted inversion recovery spin echo (DW-IR-SE) sequence with spiral readouts at ultra-strong gradients to acquire a rich diffusion–relaxation data set with sensitivity to myelin water. We reconstruct 1D and 2D spectra with a two-step convex optimization approach and investigate a variety of multidimensional MRI methods, including 1D multi-component relaxometry, 1D multi-component diffusometry, 2D relaxation correlation imaging, and 2D diffusion-relaxation correlation spectroscopic imaging (DR-CSI), in terms of their potential to quantify tissue microstructure, including the myelin water fraction (MWF). We observe a distinct spectral peak that we attribute to myelin water in multi-component T1 relaxometry, T1-T2 correlation, T1-D correlation, and T2-D correlation imaging. Due to lower achievable echo times compared to diffusometry, MWF maps from relaxometry have higher quality. Whilst 1D multi-component T1 data allows much faster myelin mapping, 2D approaches could offer unique insights into tissue microstructure and especially myelin diffusion.

## Introduction

Magnetic resonance imaging (MRI) is indispensable in neuroimaging. However, its resolution is limited and information about cellular microstructure, e.g. the amount of healthy myelin, can only be gathered indirectly. This information would be crucial to detect and monitor neurodegenerative diseases, e.g. demyelination in Multiple Sclerosis (MS). Myelin mapping can not only be used for the diagnosis of such diseases, but can also provide clinicians with further information that is not available from current clinical routine protocols, like T1-weighted imaging, T2-weighted imaging and fluid-attenuated inversion recovery (FLAIR). Whilst MS lesions may be visible in those contrasts and parametric maps, myelin content can additionally be used to e.g. distinguish MS lesions by age, or distinguish demyelination, re-myelination, edema, and other forms of neuroinflammation in a number of diseases. Beyond visible lesions, also normal-appearing white matter is known to show decreased myelin content in patients with MS [36, 40]. Further, the use of quantitative values is valuable in comparisons across different scan methods and subjects, or in longitudinal studies. To this end, researchers have been working on the quantification of myelin content via non-invasive MRI methods in the central nervous system for decades [39, 56].

A number of studies demonstrated promising results for the detection of myelin itself [28, 58, 60], but most research concentrates on the detection of water within the myelin layers and mapping of the so-called myelin water fraction (MWF) [2, 36, 40]. Methods of sensitizing the experiments to the myelin water signal include multi-exponential T2 decays (multiple spin echos or GRASE) [20, 22, 29, 39, 49], T1 relaxation (multi-inversion recovery (IR) data or ViSTa) [33, 47, 54], T2 preparation [44–46], and flip angle variation in the steady state (mcDESPOT) [18].

Disentangling the MWF signal from other water pools from a multi-exponential decay still remains challenging. Parametric approaches assume a given biophysical model, i.e. a given number of water pools (usually two or three) [14, 15, 22, 34], whilst non-parametric approaches gain comprehensive microstructure information without confining the solution to a certain model. To that end, spectra of tissue parameters like T1, T2 or diffusivity D are reconstructed—either in one or more dimensions. Early non-parametric studies focussed on nuclear magnetic resonance (NMR) experiments in phantoms, ex vivo tissue, and water in solids like wood or stone [21, 26, 42, 53, 59], whilst such demanding experiments were not yet feasible for in-vivo studies on clinical MRI systems.

More recently, several techniques for regularizing these ill-conditioned inverse problems in various tissues emerged. Zhang and Blümich combined high-resolution images and low-resolution T2-diffusion correlation data to improve the signal-to-noise ratio (SNR) in correlation imaging [64]. Benjamini et al. first solved two 1D problems separately and then used the solutions as constraints to solve the 2D correlation problem [6, 7]. Kim et al. used spatial regularization in T1-T2 correlation in vivo, i.e. penalize differences between neighboring spectra [31, 32]. Zhang et al. used a spatial total variation constraint [65]. Almeida-Martins et al., Reymbaut et al., and Martin et al. increased the dimensionality to 5D and 6D correlation MRI, by additionally varying the shape of b-tensors to encode diffusion and solving the inverse problem with a Monte Carlo-inversion [1, 41, 51]. Zibetti et al. jointly solved image reconstruction and multi-component relaxometry, leveraging sparsity and spatial correlation [66]. Avram et al. used voxel-wise optimized L2 regularization for T1-D correlation in vivo [5]. Canales-Rodríguez et al. compared several methods for solving multi-component T2 relaxometry in vivo [12, 13]. Yu et al. and Endt et al. showed promising in vivo results using supervised deep learning in 1D and 2D relaxation correlation MRI, respectively [24, 25, 63], whilst Slator et al. combined spatial information with unsupervised deep learning to solve 2D correlation imaging [52].

Whilst some of the mentioned multidimensional relaxometry and correlation imaging studies were able to detect the myelin water compartment, others did not achieve this. We tackle the challenge of myelin water’s fast T2 relaxation with a combination of spiral readouts and strong gradients of up to $300\,\hbox {mT}\,\hbox {m}^{-1}$ to achieve short echo times *TE* in a diffusion-weighted inversion recovery spin echo (DW-IR-SE) sequence [43], whilst ensuring high image quality with a reconstruction that accounts for spatio-temporal field dynamics in an advanced encoding model [62]. This allows us to acquire a rich data set with a sensitivity to T1, T2, diffusion, or combinations thereof, and investigate the potential of different 1D and 2D variants of 1D multi-component relaxometry, 1D multi-component diffusion imaging, 2D relaxation correlation imaging, and 2D diffusion–relaxation correlation spectroscopic imaging (DR-CSI) for the quantification of brain microstructure and especially myelin water mapping.

## Theory

Multi-component MRI assumes that the measured transverse magnetization *M* originates from different compartments, characterized by different T1, T2 and diffusivity D (here used synonymously for the apparent diffusion coefficient (ADC) and the mean diffusivity (MD)). Different voxels *r* are made up of a different compartment mixture, characterized by each compartment’s signal fraction *f*. Based on the known exponential equations for relaxation and diffusion in a DW-IR-SE sequence, and the scan parameters inversion time *TI*, echo time *TE* and b-value *b*, which make the scan sensitive to T1, T2 and D, respectively, the signal *M* at position *r* is described by the weighted sum

1 $$\begin{aligned} \begin{aligned} M_p(r)&\propto \int _{{\textrm{T1}},{\textrm{T2}},{\textrm{D}}} f(r,{\textrm{T1}},{\textrm{T2}},D) \left( 1 - 2\exp \left( -\frac{TI_p}{{\textrm{T1}}}\right) + \exp \left( -\frac{TR}{{\textrm{T1}}}\right) \right) \\&\quad \exp \left( -\frac{TE_p}{{\textrm{T2}}}\right) \exp \left( -b_pD\right) {\textrm{dT1}}\,{\textrm{dT2}}\,{\textrm{dD}}, \end{aligned} \end{aligned}$$

where we number a total of *P* sets of scan parameters (different *TI*, *TE*, or *b*) with the index *p*. Although non-parametric approaches do not assume a biophysical model with a defined set of water pools, we still need to discretize Eq. 1 in order to quantify the compartments. Assuming *Q* unique combinations of tissue parameters T1, T2 and D (each defining a possible component), we get:

2 $$\begin{aligned} \begin{aligned} M_p(r)&\propto \sum _{q=1}^Q f_q(r) \left( 1 - 2\exp \left( -\frac{TI_p}{{\textrm{T1}}_{q}}\right) + \exp \left( -\frac{TR}{{\textrm{T1}}_{q}}\right) \right) \\&\quad \exp \left( -\frac{TE_p}{{\textrm{T2}}_{q}}\right) \exp \left( -b_p{\textrm{D}}_q\right) . \end{aligned} \end{aligned}$$

Pre-computation of a kernel *K*

3 $$\begin{aligned} \begin{aligned} K(p,q)&= \left( 1 - 2\exp \left( -\frac{TI_p}{{\textrm{T1}}_{q}}\right) + \exp \left( -\frac{TR}{{\textrm{T1}}_{q}}\right) \right) \\&\quad \exp \left( -\frac{TE_p}{{\textrm{T2}}_{q}}\right) \exp \left( -b_p{\textrm{D}}_q\right) \end{aligned} \end{aligned}$$

of dimensions $P\times {}Q$, which contains the exponential decays for all combinations of imaging and tissue parameters, allows the forward problem of simulating the signals *M* to be formulated as a matrix multiplication:

4 $$\begin{aligned} M^{N\times {}P} = K^{P\times {}Q}F^{Q\times {}N}, \end{aligned}$$

where *N* is the number of voxels. For every voxel, the spectrum *F* contains the signal fractions for all *Q* possible compartments. Depending on the experiment, the spectra can have one (e.g. 1D T1 spectrum) or more dimensions (e.g. 2D T1-T2 spectrum). For $P<Q$, estimating the spectra *F* from given signals is an ill-conditioned, inverse problem.

## Methods

### Data Acquisition, Reconstruction and Post-processing

In vivo brain data of three healthy volunteers were acquired on a 3T Siemens MAGNETOM Skyra Connectome MR system (Siemens Healthcare GmbH, Erlangen, Germany) with gradients of up to $300\,\hbox {mT}\,\hbox {m}^{-1}$ and a 32-channel head coil, using a custom DW-IR-SE sequence with spiral readouts [43]. All scans were performed in accordance with the local ethics board after obtaining written informed consent. For each volunteer, either *TI* and *TE*, *TI* and *b* or *TE* and *b* were varied, resulting in sensitivity to T1-T2, T1-D or T2-D, respectively. All three data sets consisted of $P=8\times 8=64$ different contrasts, i.e. different combinations of acquisition parameters *TI*, *TE*, and *b*. The different scan protocols are shown in Table 1. The maximum gradient strength used was $295\,\hbox {mT}\,\hbox {m}^{-1}$ for $b=1000\,\hbox {s}\,\hbox {mm}^{-2}$. Additionally, multi-echo Cartesian gradient echo reference scans were acquired and used for the computation of coil sensitivity and B0 maps. Field fluctuations were measured in a separate experiment with a Dynamic Field Camera (Skope Magnetic Resonance Technology AG, Zurich, Switzerland) [19].

**Fig. 1:**
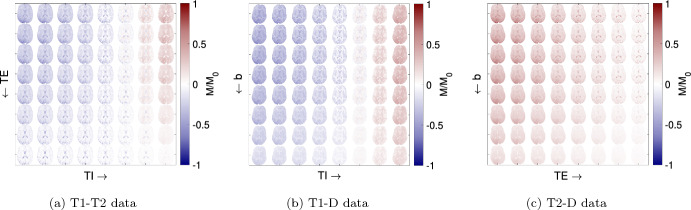
All three 2D data sets used in this study. The images show real signals *M*/$M_0$ after polarity correction and before normalization, visualizing the qualitative signal trends. After powder-averaging, each data set contains $P=8\times 8=64$ different contrasts, defined by unique combinations of acquisition parameters *TI*, *TE*, and *b*. To obtain 1D data, only the first row or column of the respective 2D data set were used. For T1-T2 and T2-D, only the 5th and 4th slice are shown, respectively

Images were then reconstructed iteratively with a conjugate gradient sensitivity encoding (SENSE) reconstruction [50]. This is based on an expanded encoding model, accounting for the measured spatio-temporal field dynamics up to third order, as well as coil sensitivities and static field inhomogeneities [61, 62], using the Skope-i software package (Skope Magnetic Resonance Technology AG, Zurich, Switzerland). Complex images were skull-stripped and registered iteratively with FSL FLIRT [30], followed by recovery of the signal polarity based on multi-exponential fits (T1-T2 and T1-D). Then, the signals obtained along the different diffusion directions were powder-averaged (T1-D and T2-D data), resulting in 64 different contrasts for all three data sets, as shown in Fig. 1. Finally, complete sets of $P=64$ signals *M* were normalized voxel-wise to their L2 norm, which removes signal offsets, leaving only the relative differences between different *TI*, *TE*, and *b*. To ensure comparability, we limit all visualizations to slices 5 and 4 for the T1-T2 and T2-D data, respectively, which show approximately the same brain structures as the T1-D data (cf. Fig. 1).

**Table 1:** Scan protocols for the DW-IR-SE acquisition of T1-T2, T1-D and T2-D data sets in individual sessions with different volunteers

	T1-T2 data	T1-D data	T2-D data
TE ($\hbox {ms}$)	4.5, 7.5, 12, 20, 33, 55, 91, 150	21	21, 28, 37, 49, 65, 86, 113, 150
TI ($\hbox {ms}$)	50, 85, 143, 243, 412, 697, 1181, 2000	50, 85, 143, 243, 412, 697, 1181, 2000	N/A
*b*-value ($\hbox {s}\,\hbox {mm}^{-2}$)	0	0, 50, 100, 200, 350, 550, 750, 1000	0, 50, 100, 200, 350, 550, 750, 1000
# diff. directions	N/A	5, 6, 6, 6, 7, 8, 10, 12	5, 6, 6, 6, 7, 8, 10, 12
TR ($\hbox {s}$)	20	5	5
Matrix	$156\times 156\times 9$	$156\times 156\times 1$	$156\times 156\times 8$
Voxel size ($\hbox {mm}^{3}$)	$1.41\times 1.41\times 5$	$1.41\times 1.41\times 4$	$1.41\times 1.41\times 4$
Volunteer	f, 32 y	f, 36 y	f, 30 y
Scan time	$21\,\hbox {min}$ $20\,\hbox {s}$	$37\,\hbox {min}$ $30\,\hbox {s}$	$37\,\hbox {min}$ $30\,\hbox {s}$

To achieve sensitivity to T1, T2 or D, the scan parameters *TI*, *TE* or *b* were varied, respectively, such that all data sets consist of $8\times 8=64$ unique contrasts

### Reconstruction of Parameter Spectra

We chose linearly spaced spectra grids with $Q_{1D}=60$ entries in 1D, or $Q_{2D}=60*60=3600$ entries in 2D, in the ranges T1$\,\in [50, 3000]\,\hbox {ms}$, T2$\,\in [5, 300]\,\hbox {ms}$, D$\,\in [0.05, 3]\,\upmu \hbox {m}^{2}\,\hbox {ms}^{-1}$. For the reconstruction of spectra, we solve a convex inverse problem in two steps with a combination of spatial regularization following Kim et al. [31, 32] and marginal constraints, inspired by Benjamini et al. [6, 7].

In the first step, we split the 2D problem into two 1D problems. To solve for one parameter (T1, T2 or D), the scan parameter encoding the other dimension (*TI*, *TE* or *b*) is kept at its minimum value (corresponding to the first row or column in Fig. 1a–c). This leaves $P_{1D}=8$ contrasts for each of the six 1D problems, reducing the unknowns-to-knowns ratio *Q*/*P* from 3600/64 in 2D to 60/8. We use the alternating direction method of multipliers (ADMM) [9, 32] to solve the following convex 1D problem:

5 $$\begin{aligned} \mathop {{\mathrm{arg\,min}}}\limits _{F\ge {}0} \sum _{i=1}^N \left( \Vert M_i-KF_i\Vert _2^2 + \lambda _{s1D}\sum _{j\in \Delta {}i}\Vert F_j-F_i\Vert _2^2\right) . \end{aligned}$$

The first term fits the signal of voxel *i* following Eq. 4. The second term serves as a regularizer and compares the solution $F_i$ in voxel *i* to its neighboring voxels *j*. The spatial regularization for the 1D problem is weighted by $\lambda _{s1D}=0.5$, which proved to stabilize the solution enough, whilst avoiding excessive smoothing.

To solve the 2D problem, we utilize a generalization of the Douglas–Rachford method [10, 11, 37, 48] and continue to use spatial regularization with a reduced weight $\lambda _{s2D}=0.01$, as we now use additional regularization:

6 $$\begin{aligned} \begin{aligned}&\mathop {{\mathrm{arg\,min}}}\limits _{F\ge {}0} \sum _{i=1}^N \left( \Vert M_i-KF_i\Vert _2^2 \right. + \lambda _{s2D}\sum _{j\in \Delta {}i}\Vert F_j-F_i\Vert _2^2 \\&\quad \left. + \lambda _m \Vert \pi _1 F_i - F_i^1\Vert _1 + \lambda _m \Vert \pi _2 F_i - F_i^2\Vert _1\right) . \end{aligned} \end{aligned}$$

The solutions of both 1D problems are considered marginals of the 2D problem and act as regularizers. The operators $\pi _{1/2}$ project the 2D solution $F_i$ of voxel *i* to its respective 1D marginal, which is compared to the corresponding 1D solutions $F_i^1$ or $F_i^2$, respectively. The marginal regularization is weighted with $\lambda _m=10^3$. This keeps the marginals of the 2D solution close to the solution of the much less ill-conditioned 1D problem, whilst the distribution within the 2D space can still be diversified. Finally, spectra are averaged over the whole slice to identify sub-compartments and delineate them with thresholds.

### Signal Fractions and Visualization

Compartmental signal fractions maps were computed by thresholded integration of the voxel-wise spectra. The signal fraction of myelin water is computed as follows:

7 $$\begin{aligned} {\textrm{MWF}}({\textbf{r}}) = \int _{{\textrm{T1}}_{\textrm{min}}^{\textrm{MWF}}}^{{\textrm{T1}}_{\textrm{max}}^{\textrm{MWF}}}\int _{{\textrm{T2}}_{\textrm{min}}^{\textrm{MWF}}}^{{\textrm{T2}}_{\textrm{max}}^{\textrm{MWF}}}F({\textbf{r}},{\textrm{T1}},{\mathrm {{T2}}}){\textrm{dT1}}{\textrm{dT2}} \end{aligned}$$

for the T1-T2 case. For other compartments or spectra in both 1D and 2D, the equation can be formulated analogous. In all cases, the compartmental signal fractions are normalized to

8 $$\begin{aligned} {\textrm{MWF}}+f_{\mathrm {IC/EC}}+f_{\textrm{CSF}} \overset{!}{=}\ 1. \end{aligned}$$

For consistency with literature, we use the abbreviation MWF, which is equivalent to $f_{\textrm{MW}}$. For the visualization of 2D spectra and compartmental signal fraction maps, we use Scientific colour maps by Crameri et al. [16, 17].

Further, the spectra are used to simulate predicted signal evolutions, following Eq. 4, which are then compared to the actual data by computing the root-mean-square error (RMSE).

## Results

**Fig. 2:**
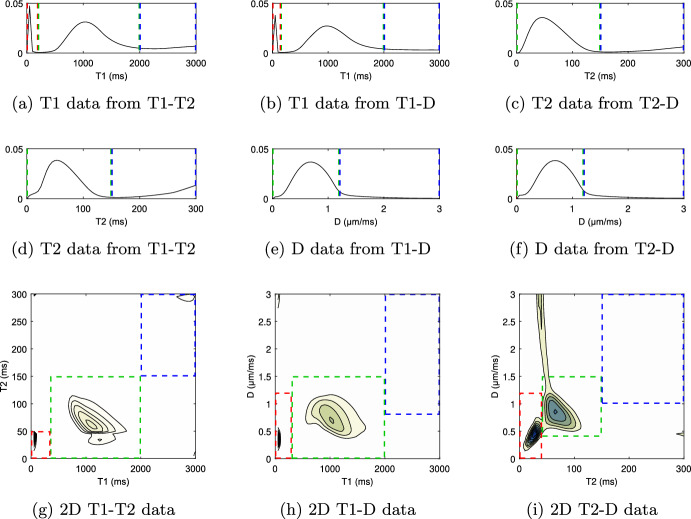
Resulting spectra for all three data sets. **a**–**f** show the 1D spectra for the different sub-sets of our data. **g**–**i** show the 2D correlation spectra using all of the respective data sets. Dashed lines and rectangles indicate the thresholds used to define the different sub-compartments. Red: fast relaxing, only visible in **a**,**b** and **g**–**i**, attributed to myelin water. Green: Medium fast relaxing, attributed to IC/EC. Blue: slow relaxing, attributed to CSF

Figure 2 shows the full spectra for T1-T2, T1-D, T2-D, and their respective 1D subsets, averaged over the whole slice. We find three distinct spectral compartments in 1D T1 and 2D data, and two compartments in 1D T2 and 1D diffusion spectra. We attribute these spectral components to the following microstructure compartments (low to high T1/T2/D): Myelin water at T1$=50\,\hbox {ms}$, T2$\approx 20-45\,\hbox {ms}$, D$\approx 0.3- 0.55\,\upmu \hbox {m}^{2}\,\hbox {ms}^{-1}$, combined intra- and extra-cellular space (IC/EC) (a broader peak at T1$\approx 700-1400\,\hbox {ms}$, T2$\approx 50-95\,\hbox {ms}$, D$\approx 0.5- 1.1\,\upmu \hbox {m}^{2}\,\hbox {ms}^{-1}$) and cerebrospinal fluid (CSF). The MWF peak is not visible in 1D T2 and 1D diffusion spectra. The CSF compartment is very broad but present at high T1/T2/D. Dashed lines and rectangles indicate manually set thresholds that define the sub-compartments. For 2D spectra, especially for T2-D, there are some spurious signals outside of the delineated areas, which we ignore for further analysis.

**Fig. 3:**
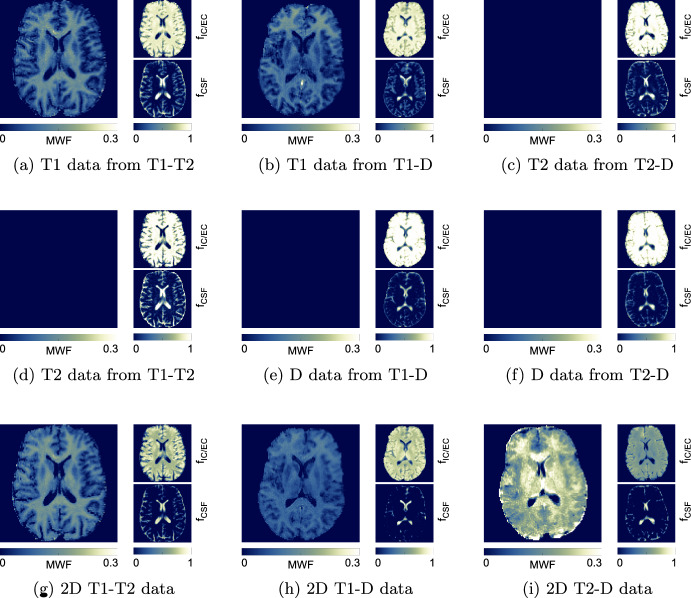
Compartmental signal fractions spectra for all three data sets, as they result from the thresholded integration of *F*. **a**–**f** use the different 1D subsets of our data, whilst **g**–**i** show the 2D solutions using all data. In each sub-figure, the myelin water sub-compartment (MWF) is shown enlarged to the left, followed by IC/EC and CSF to the right. A distinctive MWF peak was only identified in five methods, resulting in a MWF of 0 in subfigures **c**–**f**. Note that we chose a different color bar scaling for the MWF map

Figure 3 shows the resulting compartmental signal fraction maps for all three sub-compartments, as obtained from all nine spectra. The MWF map is only present where previously delineated.

**Fig. 4:**
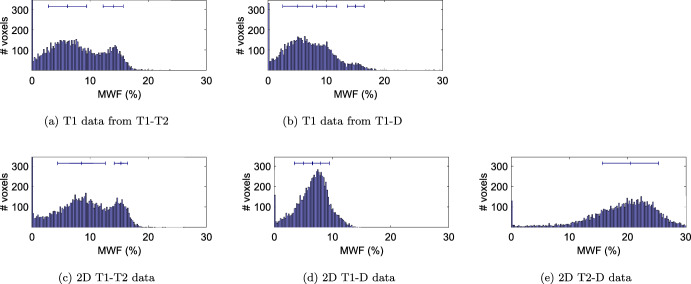
Histogram of MWF values for all five methods that were able to reconstruct a distinct MWF peak. The bars above the histograms show the mean and standard deviation of fitted Gaussians. In sub-figures **a**–**c**, two distinctive peaks are visible, which can be attributed to gray matter and white matter. **b** shows an additional third peak at higher MWF which is present in WM of the frontal lobe. In sub-figure (**d**), GM and WM do not show distinct peaks, but are still separable, whilst in sub-figure (**e**) we could fit only one Gaussian for both. The peaks at $0\%$ are attributed to CSF. Histogram bin width is $0.2\%$

Histograms of these five MWF maps (Fig. 4) show similar myelin water fractions for 1D T1 from T1-T2, 1D T1 from T1-D, and 2D T1-T2 data. The data show two peaks, which can be attributed to gray matter (GM) and white matter (WM), respectively. Data from 2D T1-D and 2D T2-D also show higher values in WM than GM, but the peaks are not as well separated. Gaussian fits to the histograms yield the following quantitative values:

- 1D T1 from T1-T2: MWF of $\approx 6.2\%\pm 0.3\%$ in GM and $\approx 14.0\%\pm 1.7\%$ in WM,
- 1D T1 from T1-D: MWF of $\approx 5.1\%\pm 2.6\%$ in GM and $\approx 10.1\%\pm 1.7\%$ in WM, with values $\approx 15.1\%\pm 1.4\%$ in the frontal lobe, which is also visible in Fig. 3 b),
- 2D T1-T2: MWF of $\approx 8.5\%\pm 4.1\%$ in GM and $\approx 15.3\%\pm 1.2\%$ in WM,
- 2D T1-D: MWF of $\approx 5.1\%\pm 1.6\%$ in GM and $\approx 8.0\%\pm 1.5\%$ in WM,
- 2D T2-D: MWF of $\approx 20.4\%\pm 4.8\%$, with GM and WM being hard to separate.

In CSF, we find MWF values of $\approx 0\%$ for 1D T1 from T1-T2, 1D T1 from T1-D, and 2D T1-T2 data. In both 2D T1-D and 2D T2-D data, the algorithm yield a false positive MWF of $\approx 1\%$ in CSF.

**Fig. 5:**
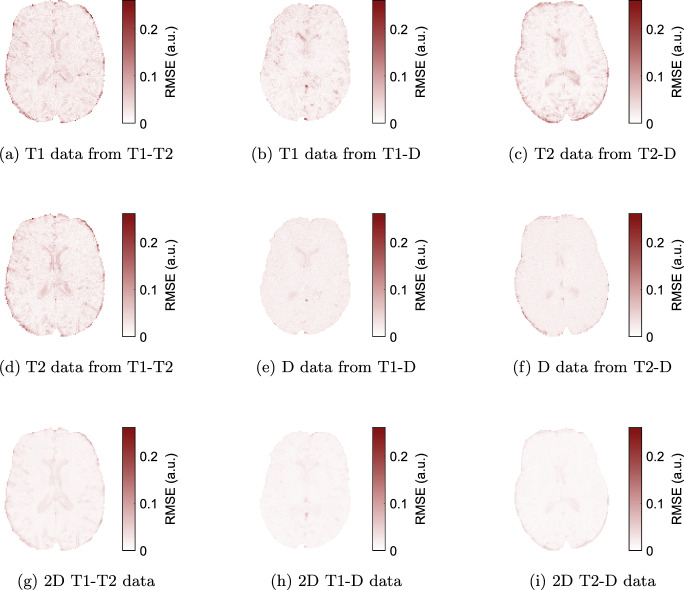
Root-mean-square error (RMSE) of signal fits simulated from the spectra compared to the original signals for all data sets. Subfigures **a**–**f** show the multi-component 1D methods, **g**–**i** show the 2D methods

## Discussion

Our scan protocols were designed for brain microstructure quantification with a focus on myelin water. Using spiral readouts and strong diffusion gradients up to $300\,\hbox {mT}\,\hbox {m}^{-1}$, we achieved a minimum *TE* of $4.5\,\hbox {ms}$ without diffusion weighting and $21\,\hbox {ms}$ with diffusion weighting of up to $b=1000\,\hbox {s}\,\hbox {mm}^{-2}$ (cf. Table 1). Whilst these gradients are not the clinical standard, the T1-T2 data does not rely on them and can just as well be acquired on clinical systems. With limited scan times and to achieve sufficiently fine spectra gridding at lower values, we did not include any $TE>150\,\hbox {ms}$ and chose to reconstruct spectra with a maximum T2 of $300\,\hbox {ms}$. Therefore, the capability to estimate T2 is limited in CSF, as shown in the error maps (Fig. 5).

Two compartments were found in all spectra and can be attributed to the combined intra- and extra-cellular space (IC/EC), and CSF (Fig. 2). A third, fast relaxing compartment was found in five out of nine slice-average spectra. This compartment was identified as myelin water. The resulting MWF maps (Fig. 3) from 1D T1 data at $TE=4.5\,\hbox {ms}$ (subset of T1-T2 data) and from 2D T1-T2 data show high quality and have good WM-GM contrast. MWF from T1 data at $TE=21\,\hbox {ms}$ (subset of T1-D data) has inferior WM-GM contrast and an implausible MWF increase in the whole frontal lobe. Whilst exhibiting good image quality, the MWF map obtained from 2D T1-D data shows the lowest values and has a worse WM-GM contrast. For 2D T2-D data, the MWF map quality is not acceptable with the highest MWF values and little WM-GM contrast.

The quantitative MWF peaks around $5--8\%$ in GM and $8--15\%$ in WM, excluding the T2-D data. These values lie within most ranges reported in the literature [4, 23, 27, 35, 38, 39, 46, 55, 57, 63]. However, some studies report higher values in WM [8]. Apart from comparisons with different in vivo methods, the results should also be validated with histological examinations. Whilst this is beyond the scope of this study, a few previous studies showed good agreement of similar methods with ex-vivo histology of different tissues [63, 65]. This includes the work of Benjamini et al., which inspired the design of our regularized spectra reconstruction framework [7]. The width of the MWF distributions (Fig. 4) beyond the standard deviation of fitted Gaussians is attributed to partial volumes and noise. We also observed slightly higher MWF in inner parts of WM structures, compared to outer parts.

A myelin water T2 around $\approx 30\,\hbox {ms}$ leaves us with less than $60\%$ of the myelin water signal in any diffusion data ($TE\ge 21\,\hbox {ms}$) compared to $TE=4.5\,\hbox {ms}$. This may explain the lower MWF map quality in diffusion experiments and makes the results in 1D T1 spectra, acquired at $TE=4.5\,\hbox {ms}$, and 2D T1-T2 spectra the most promising for MWF mapping in this study. These findings confirm that the low *TE* achieved with spiral readouts are crucial for MWF mapping, whilst diffusion weighting can be a limiting factor. We did not find a distinct myelin water peak in 1D T2 spectra. However, previous research did achieve the reconstruction of MWF maps from 1D T2 relaxometry. The main difference could be that with 32 echo times, these studies used four times the amount of data [12, 13, 63]. Our work shows that multi-component T1 relaxometry in 1D may allow MWF mapping of comparable quality to 2D correlation imaging in a fraction of the scan time—using only 8 inversion times.

Whilst the incorporation of diffusion weighting prolongs the echo times, diffusion–relaxation correlation imaging in 2D is promising for the measurement of myelin water’s diffusion coefficient. Our results for the mean diffusivity are in reasonable agreement with previous research, although there is a notable directional dependence of D within in myelin water [3]. For the present work, we used a powder-average of the directional diffusion information, as we aim to compare 1D and 2D approaches with the same number of signals ($P_{1D}=8$, $P_{2D}=64$). In the future, the explicit use of directional diffusion information could benefit the reconstruction and is subject to ongoing work. Further, we envision that our information on myelin water diffusion, combined with directional information will be useful for the investigation of e.g. neurodevelopment, ageing, or to differentiate demyelination from re-myelination.

The RMSE maps in Fig. 5 consistently show that there is a benefit of 2D correlation imaging compared to 1D multi-component approaches, although the 2D data partly contain more noise at higher *TE* and *b*. Further, 2D approaches are more likely to reliably separate IC and EC compartments in the future.

The three different data sets were acquired in different volunteers. However, we chose healthy volunteers of the same gender and similar age (cf. Table 1), and similar slice positioning, avoiding the most prominent sources of MWF differences across different subjects [4, 27]. The assessment of both reproducibility in more volunteers and of clinical relevance is subject to future work.

Once properly trained, neutral networks are promising for the reconstruction of both 1D and 2D spectra [24, 25, 52, 63]. However, the hyper-parameter optimization and training have to be repeated for every data set and would be extremely time-consuming for a broad comparison of data sets as in this study.

## Conclusion

In our work comparing 1D and 2D methods, we demonstrate how diffusion–relaxation correlation MRI can enable in vivo myelin water quantification. To this end, we investigated 1D multicomponent relaxometry, 1D multicomponent diffusion imaging, 2D relaxation correlation imaging, and 2D diffusion–relaxation correlation spectroscopic imaging (DR-CSI).

Using a DW-IR-SE sequence on a 3T Connectome MR system, we acquired a rich data set with sensitivity to T1, T2 and diffusion in healthy volunteers. Spiral readouts and ultra-strong gradients achieve *TE* as low as $4.5\,\hbox {ms}$ without and $21\,\hbox {ms}$ with diffusion encoding up to $b=1000\,\hbox {s}\,\hbox {mm}^{-2}$, whilst ensuring high image quality with an image reconstruction based on an expanded encoding model.

Expanding previous reconstruction approaches for 2D correlation spectra with a two-step convex optimization, we demonstrated the successful reconstruction of both 1D and 2D spectra in brain in vivo. Both 1D T1 spectra and all 2D spectra (T1-T2, T1-D, T2-D) showed a distinct myelin water signal peak and thus enable the study of myelin in vivo. Consistently, the solutions found for the 2D correlation experiments depict lower errors than their respective 1D marginals. We achieve the best qualitative results for T1-T2 data, but given advances to similarly short *TE* in diffusion-weighted imaging, myelin correlation imaging could increasingly benefit from the unique qualities of diffusion MRI in the future.

As compared to prior work oftentimes focusing on T1-T2 correlation, our approach to include diffusion encoding in the 2D correlation experiments and to develop a consistent framework for robust solutions of the ill-posed inversion problem will in the future enable the study of myelin in greater detail.

## Data Availability

The data sets generated and analysed during the current study are not publicly available as the participants did not give consent for the data to be shared publicly, but the data sets are available from the corresponding author on reasonable request.
